# Revealing Beta-Diversity Patterns of Breeding Bird and Lizard Communities on Inundated Land-Bridge Islands by Separating the Turnover and Nestedness Components

**DOI:** 10.1371/journal.pone.0127692

**Published:** 2015-05-18

**Authors:** Xingfeng Si, Andrés Baselga, Ping Ding

**Affiliations:** 1 College of Life Sciences, Zhejiang University, Hangzhou, Zhejiang, 310058, China; 2 Departamento de Zoología, Facultad de Biología, Universidad de Santiago de Compostela, c/Lope Gómez de Marzoa s/n, 15782, Santiago de Compostela, Spain; University of Brasilia, BRAZIL

## Abstract

Beta diversity describes changes in species composition among sites in a region and has particular relevance for explaining ecological patterns in fragmented habitats. However, it is difficult to reveal the mechanisms if broad sense beta-diversity indices (i.e. yielding identical values under nestedness and species replacement) are used. Partitioning beta diversity into turnover (caused by species replacement from site to site) and nestedness-resultant components (caused by nested species losses) could provide a unique way to understand the variation of species composition in fragmented habitats. Here, we collected occupancy data of breeding birds and lizards on land-bridge islands in an inundated lake in eastern China. We decomposed beta diversity of breeding bird and lizard communities into spatial turnover and nestedness-resultant components to assess their relative contributions and respective relationships to differences in island area, isolation, and habitat richness. Our results showed that spatial turnover contributed more to beta diversity than the nestedness-resultant component. The degree of isolation had no significant effect on overall beta diversity or its components, neither for breeding birds nor for lizards. In turn, in both groups the nestedness-resultant component increased with larger differences in island area and habitat richness, respectively, while turnover component decreased with them. The major difference among birds and lizards was a higher relevance of nestedness-resultant dissimilarity in lizards, suggesting that they are more prone to local extinctions derived from habitat fragmentation. The dominance of the spatial turnover component of beta diversity suggests that all islands have potential conservation value for breeding bird and lizard communities.

## Introduction

Beta diversity, or the amount of change in species composition among sites in a region [1], has particular relevance for explaining ecological patterns in regional biodiversity [2–4]. Numerous dissimilarity indices have been proposed in the literature [5–7] to quantify the variation in species composition among sites. All these indices aim to infer the mechanisms behind variation in species composition [8]. However, it has been shown that broad sense dissimilarity indices yield identical results under different patterns deriving from different ecological processes [9–11]. In particular, two antithetic processes both contribute to beta diversity [2, 12]: species replacement (spatial turnover) and species richness differences, which may be between nested or not nested assemblages [9, 13–15]. It is thus critical the need to partition beta diversity into separate components accounting for these patterns, because confusing two antithetic phenomena as being a single pattern could prevent understanding the ecological processes behind the observed patterns [9, 16]. As a consequence, partitioning beta diversity will provide a unique way to understand the variation of species composition among sites, with interest for basic biogeography and ecological applications [17–19].

Recently, Baselga [9] proposed that Sørensen dissimilarity [20], a monotonic transformation of strict sense beta diversity (i.e. gamma/alpha), could be decomposed into two additive components accounting for spatial turnover and nestedness-resultant dissimilarities, respectively. Spatial turnover is caused by the replacement of species from one site to another, which may be the result of niche and dispersal processes, either contemporary or historical [11, 16, 21, 22]. Contrary to turnover, the nestedness-resultant component is determined by species loss or gain in nested subsets, which may be due to contemporary or historical processes as selective extinction, selective colonization, habitat nestedness [4, 16, 23–25]. Thus, separating both components of dissimilarity can help to unveil the ecological processes [26, 27]. For example, Baselga et al. [28] found no clear latitudinal gradient in large-scale beta-diversity patterns of world amphibians. However, when both components were separated, clear latitudinal gradients were observed, with spatial turnover dominating low latitudes and nestedness-resultant dissimilarity being more relevant at high latitudes, pointing to marked differences in the processes driving beta diversity at low and high latitudes (see also [24]). Besides its interest for basic biogeographical and ecological questions, quantifying the proportion of each component of beta diversity is also crucial for planning conservation strategies [22]. If nestedness contributes more than turnover into overall beta diversity among sites, it might suggest that sites with richer in species should be prioritized for protection. On the other hand, if the spatial turnover component is the dominant phenomenon, all sites should be potential targets for conservation [11]. As a result, many studies applied methods for partitioning beta diversity to delineate conservation strategies for various taxonomic groups [4, 29, 30].

Habitat fragmentation caused by human activities compromises the conservation of biological communities. Biodiversity falls rapidly under habitat fragmentation because species are prone to local extinction due to habitat loss, and thus animal communities in small habitats have higher extinction vulnerability [31, 32]. In consequence, small habitat patches are generally considered of low conservation priority and they are given little protection [33–35]. Meanwhile, fragmentation increases beta diversity by creating patchiness in species distributions because of differential local extinction of species among fragments [36]. Therefore, fragmentation represents a challenge for conservation, because the remaining small fragments may not be sufficient to support viable populations, but still make a substantial contribution to regional diversity [3, 37]. In this way, small remnants of fragment habitats may have potential conservation value that should not be overlooked [37]. It is thus relevant to know whether compositional differences between fragments (i.e. beta diversity) are related to replacement and/or nested patterns. Although numerous studies have addressed the effects of fragmentation on species communities (e.g. [38–41]), few studies have used beta-diversity partitioning for species communities in fragmented habitats, especially comparing data on multiple taxonomic groups [42, 43].

Here, we study beta-diversity patterns of breeding bird and lizard communities on islands in an inundated lake in eastern China by partitioning overall dissimilarity among islands into turnover and nestedness-resultant components. We collected occupancy data on 37 islands for birds (from 2007 to 2012) and for lizards (from 2007 to 2008). We used these data to assess the spatial patterns of beta diversity and its components, and address the following questions: (1) Are beta diversity and its components different between breeding bird and lizard communities? (2) Which component, turnover or nestedness-resultant dissimilarity, contribute more to overall beta diversity of both groups? (3) What are the relationships between beta diversity components and differences in island attributes? (4) Based on the relative contributions of turnover and nestedness-resultant components, what are the potential conservation strategies for biodiversity management on land-bridge islands, such as the systems inundated recently? Our initial expectations were that birds generally have lower overall beta diversity, especially the nestedness-resultant component, and thus a lower contribution of nestedness-resultant component into overall beta diversity because of its higher vagility [4, 24, 44].

## Materials and Methods

### Ethics Statement

Our research on breeding bird and lizard communities in the Thousand Island Lake was approved by the Chinese Wildlife Management Authority and conducted under Law of the Peoples Republic of China on the Protection of Wildlife (August 28, 2004). Chun’an Forestry Bureau and the Thousand Island Lake National Forest Park granted the permits to conduct the researches.

### Study Area

**Fig 1:**
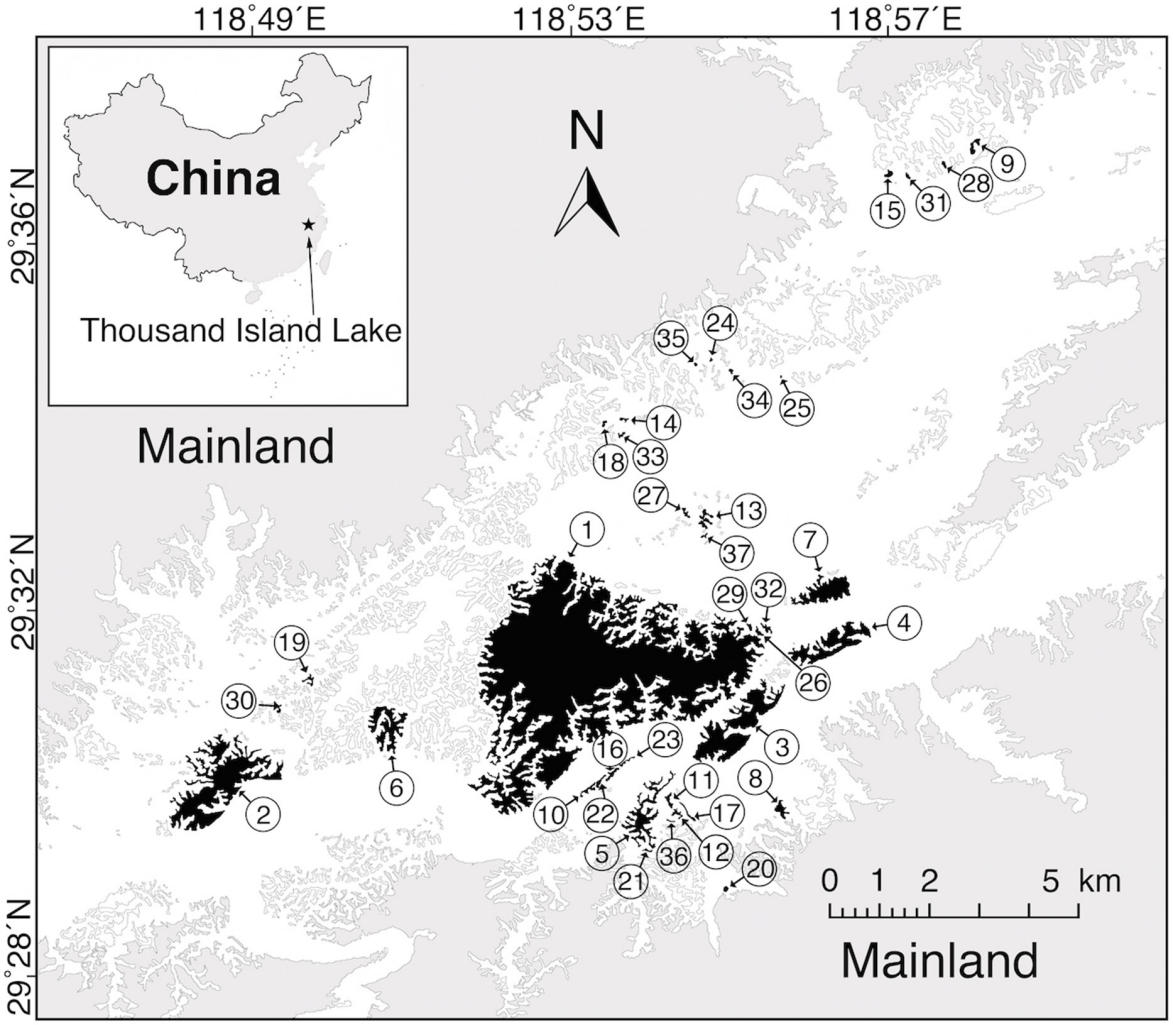
The 37 study islands in the Thousand Island Lake in Zhejiang Province, China. Study islands were numbered in order of decreasing area.

Thousand Island Lake lies at 29°22´ to 29°50´ N, 118°34´ to 119°15´ E and is a large artificial reservoir in western Zhejiang Province, eastern China (Fig 1). The lake was created in 1959 by the construction of the Xin’anjiang Dam for hydroelectricity. Flooding approximately 580 km^2^, it formed 1078 islands with areas > 0.25 ha when the water reached its highest level (108 m). The dominant vegetation on the islands is natural secondary forest, mainly of *Pinus massoniana*, with many broad-leaved trees and shrub species, such as *Castanopsis sclerophylla*, *Liquidambar formosana*, *Rhododendron farrerae*, and *Loropetalum chinense*. The region has a typical subtropical monsoon zone, with marked seasonality. The average annual temperature is 17.0°C. Daily temperature ranges from—7.6°C in January to 41.8°C in July. The annual precipitation of the region is 1430 mm, mainly concentrated in rainy season between April and June [43].

Land-bridge islands created by construction of dams, such as the islands of the Thousand Island Lake, can be viewed as natural experiments for assessing the effects of fragmentation on community composition variation [45]. Our study system is particularly well suited for two reasons. First, the islands were created essentially simultaneously as a result of dam construction and the quick subsequent inundation, so that all islands have the same ecological background and clear geological boundaries [46, 47]. Moreover, given the short history of the lake (55 years), all the islands still share an ancestral pool of species [48], and the effects of long-term historical processes (e.g. glacial cycles and speciation) can be excluded [16, 24, 25]. Second, the islands are relatively small and accessible, so we were able to survey the animal communities on islands thoroughly several times in each year, providing a relatively complete animal inventory in our research system.

### Sampling Protocols

#### Island Attributes

We selected 37 islands (numbered from largest to smallest according to island area in Fig 1) to encompass as much variation in area and isolation as possible. These islands range from 0.57 ha to approximately 1300 ha, and from about 20 m separation from the nearest shore of the mainland to over 3.71 km. We characterized islands in terms of area, isolation and habitat richness, as these variables are recognized as the key determinants of the probabilities of colonization and extinction [49, 50], thus being potentially relevant factors behind beta diversity and its components [51].

For each island, we measured area in hectares (*A*). For isolation measures, we used a buffer-based measure to estimate the isolation, which is generally considered better than distance-based measures [52]. We estimated isolation (*I*) as the fraction of buffer area that is water within a 2-km buffer region around a focal island [48]. Between April and November in 2007, we intensively surveyed study islands, and classified the habitats on each island into seven categories: coniferous forest, broad-leaved forest, mixed coniferous broad-leaf forests, bamboo groves, shrubs, grasses and farmland [43]. We then defined habitat richness (*Hr*) as the number of habitat types on each island (Table 1).

**Table 1:** Characteristics of the 37 study islands in the Thousand Island Lake, China.

Island code	Latitude	Longitude	Island area (ha)	Isolation	Species richness of birds (n)	Species richness of lizards (n)	Habitat richness (n)	Number of transects (n)	Total length of transects (m)
1	29°31´11.44´´N	118°52´25.87´´E	1289.23	0.78	43	5	7	8	3200
2	29°30´30.15´´N	118°49´09.31´´E	143.19	0.88	34	4	6	4	1600
3	29°31´09.85´´N	118°55´15.92´´E	109.03	0.73	35	4	6	4	1600
4	29°31´51.52´´N	118°56´24.47´´E	55.08	0.79	32	4	5	2	800
5	29°29´40.04´´N	118°53´39.07´´E	46.37	0.68	31	3	5	2	800
6	29°30´52.90´´N	118°50´57.48´´E	35.64	0.63	27	4	5	2	800
7	29°32´06.77´´N	118°56´13.82´´E	32.29	0.88	30	3	5	2	800
8	29°29´45.04´´N	118°55´42.22´´E	5.69	0.57	30	2	3	1	375
9	29°37´07.60´´N	118°58´03.22´´E	3.42	0.69	24	0	4	1	300
10	29°30´01.91´´N	118°53´08.99´´E	2.90	0.67	20	1	3	1	275
11	29°29´54.89´´N	118°54´13.92´´E	2.83	0.77	25	2	4	1	150
12	29°29´45.77´´N	118°54´22.48´´E	2.29	0.77	26	3	4	1	300
13	29°32´58.32´´N	118°54´37.70´´E	2.23	0.98	23	0	3	1	400
14	29°34´03.33´´N	118°53´42.12´´E	2.00	0.74	23	2	3	1	300
15	29°36´45.05´´N	118°57´00.39´´E	1.93	0.73	24	1	4	1	250
16	29°30´12.53´´N	118°53´31.07´´E	1.74	0.66	20	2	3	1	300
17	29°29´43.37´´N	118°54´33.45´´E	1.54	0.76	25	1	3	1	375
18	29°30´28.07´´N	118°49´24.48´´E	1.52	0.71	24	1	3	1	250
19	29°31´14.60´´N	118°49´38.74´´E	1.40	0.68	23	1	3	1	375
20	29°28´55.79´´N	118°54´55.49´´E	1.26	0.42	25	1	3	1	200
21	29°29´24.45´´N	118°54´01.52´´E	1.20	0.67	22	1	3	1	225
22	29°30´11.27´´N	118°53´25.38´´E	1.20	0.67	23	2	3	1	225
23	29°30´19.16´´N	118°53´38.69´´E	1.17	0.63	20	2	3	1	250
24	29°34´47.56´´N	118°54´43.16´´E	1.15	0.67	23	0	3	1	275
25	29°34´36.77´´N	118°55´38.49´´E	1.03	0.80	18	1	3	1	250
26	29°31´45.45´´N	118°55´21.56´´E	1.01	0.72	24	1	3	1	250
27	29°33´06.81´´N	118°54´26.16´´E	0.96	0.98	19	0	3	1	250
28	29°36´51.21´´N	118°57´43.00´´E	0.91	0.74	23	0	4	1	275
29	29°31´48.11´´N	118°55´18.05´´E	0.86	0.70	22	1	3	1	225
30	29°30´54.55´´N	118°49´21.12´´E	0.83	0.74	22	1	3	1	275
31	29°36´48.30´´N	118°57´12.92´´E	0.83	0.75	20	0	4	1	250
32	29°31´47.12´´N	118°55´28.34´´E	0.80	0.74	25	1	2	1	300
33	29°33´58.20´´N	118°53´43.87´´E	0.73	0.80	23	2	3	1	300
34	29°34´38.59´´N	118°54´57.84´´E	0.67	0.75	18	1	3	1	325
35	29°34´40.16´´N	118°54´34.20´´E	0.59	0.68	25	0	3	1	225
36	29°29´42.71´´N	118°54´18.86´´E	0.59	0.78	21	1	3	1	250
37	29°32´52.20´´N	118°54´42.82´´E	0.57	0.95	19	0	3	1	200

Each island is numbered as in Fig 1. Isolation was estimated as the fraction of buffer area that is water within a 2-km buffer region around a focal island. See more details for these island attributes in the *Materials and Methods*.

#### Bird Sampling

We surveyed bird communities on 37 islands during the breeding season (April–June) annually from 2007 to 2012. The sampling effort of each island was roughly proportional to the logarithm of area [53]. As a result, eight transect trails were sampled on Island 1 (the largest study island, area > 1000 ha), four on Islands 2 and 3 (island area > 100 ha), two on four islands (10 ha < island area < 100 ha), and one on each of the remaining small islands (island area ≈ 1 ha for most islands) (Table 1). We used a global positioning system (GPS) to record the total length of transects on each island.

Transects were generally placed along ridge-lines, and we cleared narrow census trails (about 20 cm wide) to facilitate surveys [54]. Where islands had more than one habitat type, a stratified random placement was used to capture all the types. We collected bird occupancy data along these transects [55] during breeding seasons from 2007 to 2012. In each survey, observers walked each transect at a constant speed (*c*. 2.0 km × h^−1^). We recorded all the birds seen or heard on the survey island, but excluded high-flying species passing over the islands during surveys. We surveyed each transect on these islands 78 times over the course of the entire study [48]. Surveys ran from after half an hour after dawn to 11:00 h in the mornings and from 15:00 to half an hour before sunset in the afternoons. We did not conduct bird surveys if there was heavy rain, high wind, or high temperature. We alternated the direction observer walked on each transect randomly to eliminate the potential survey bias [43, 48, 56].

We assessed the completeness of our survey for the largest, and proportionally least sampled island (Island 1; see Fig 1) by creating species accumulation curves for each of the six years. We found all curves clearly leveled off before the completion of the surveys, indicating sampling efforts were sufficient on this and smaller islands to capture the full breeding bird communities [48]. In our study, we only considered terrestrial breeding birds, excluding diving birds, ducks, gulls, shorebirds, herons and kingfishers whose habitats extensively relies on water. During the course of breeding seasons from 2007 to 2012, we recorded data total of 60 terrestrial breeding birds (S1 Dataset).

#### Lizard Sampling

We used the line-transect method [57] to survey the lizard communities along transects on 37 islands that are the same as bird surveys during two breeding seasons in 2007 and 2008. Observers walked each transect at a constant speed (*c*. 10 m × min^−1^) to search the ground and tree boles with binoculars [43]. We only included the confident identifications recorded on the survey island into our analyses [58]. Surveys ran from between 1 h after sunrise until 5 h after sunrise in good weather condition. The direction observers walked on each transect and the surveyed islands were also randomly alternated.

In Zhejiang Province, there are only six lizard species [59] that are very common and easy to identify with confidence. We found all the species on the survey islands except *Takydromus sexlineatus*, whose distribution on the nearby mainland is still controversial [43]. Based on the high survey frequencies (20 times per each transect), we consider that the species lists in our dataset are complete and reliable (S2 Dataset).

### Partitioning Beta Diversity

We partitioned beta diversity into two separate components of species turnover and nestedness-resultant dissimilarities [9]. Specifically, this method partitions the pairwise Sørensen dissimilarity between two communities (β_sor_) (Eq 1) into two additive components accounting for species spatial turnover (β_sim_) (Eq 2) and nestedness-resultant dissimilarities (β_sne_) (Eq 3). The Simpson dissimilarity index (β_sim_) describes species turnover without the influence of richness gradients [5, 30, 60]. Since β_sor_ and β_sim_ are equal in the absence of nestedness, their difference is a net measure of the nestedness-resultant component of beta diversity, so that β_sne_ = β_sor_ − β_sim_ [9]. The pairwise dissimilarity indices are formulated as:

$$\beta_{sor}=\frac{b+c}{2a+b+c}$$ (1)

$$\beta_{sim}=\frac{\text{min}\left(b,c\right)}{a+\text{min}\left(b,c\right)}$$ (2)

$$\beta_{sne}=\beta_{sor}-\beta_{sim}=\frac{\left|b-c\right|}{2a+b+c}\times \frac{a}{a+\text{min}\left(b,c\right)}$$ (3)

where *a* is the number of species present at both sites, *b* is the number of species present at the first site but not at the second, and *c* is the number of species present at the second site but not at the first. The first fraction of Eq 3, $\frac{\left|b-c\right|}{2a+b+c}$, is similar as β_gl_ index, $\frac{2\max\left(b,c\right)-2min(b,c)}{2a+\max\left(b,c\right)+min(b,c)}$ [5, 13]—both of them estimate species richness differences. The second fraction of β_sne_, $\frac{a}{a+min(b,c)}$, is the Simpson similarity, 1 − β_sim_, which is a measure of nestedness [61]. As a result, β_sne_ measures the fraction of dissimilarity caused by richness differences between nested subsets [9, 15, 19].

To estimate the overall beta diversity of breeding bird communities among all islands, we used the multiple-site dissimilarity [62]. Overall multiple-site dissimilarity was measured using multiple-site Sørensen dissimilarity (β_SOR_; Eq 4), which was decomposed into spatial turnover (β_SIM_; Eq 5) and nestedness-resultant components (β_SNE_; Eq 6) [9]:

$$\beta_{SOR}=\frac{\left[\sum_{i<j}\min\left(b_{ij},b_{ji}\right)\right]+\left[\sum_{i<j}\max\left(b_{ij},b_{ji}\right)\right]}{2\left[\sum_{i}S_{i}-S_{T}\right]+\left[\sum_{i<j}\min\left(b_{ij},b_{ji}\right)\right]+\left[\sum_{i<j}\max\left(b_{ij},b_{ji}\right)\right]}$$ (4)

$$\beta_{SIM}=\frac{\left[\sum_{i<j}\min\left(b_{ij},b_{ji}\right)\right]}{\left[\sum_{i}S_{i}-S_{T}\right]+\left[\sum_{i<j}\min\left(b_{ij},b_{ji}\right)\right]}$$ (5)

$$\beta_{SNE}=\beta_{SOR}-\beta_{SIM}=\frac{\left[\sum_{i<j}\max\left(b_{ij},b_{ji}\right)\right]-\left[\sum_{i<j}\min\left(b_{ij},b_{ji}\right)\right]}{2\left[\sum_{i}S_{i}-S_{T}\right]+\left[\sum_{i<j}\min\left(b_{ij},b_{ji}\right)\right]+\left[\sum_{i<j}\max\left(b_{ij},b_{ji}\right)\right]}\times \frac{\left[\sum_{i}S_{i}-S_{T}\right]}{\left[\sum_{i}S_{i}-S_{T}\right]+\left[\sum_{i<j}\min\left(b_{ij},b_{ji}\right)\right]}$$ (6)

where *S*_*i*_ is the species richness of island *i*, *S*_*T*_ is the species richness on all study islands (γ diversity), and *b*_*ij*_ and *b*_*ji*_ are the species richness exclusive to island *i* and *j*, respectively. The fractions [Σ_*i<*_ *j* min (*b*_*ij*_,*b*_*ji*_)] and [Σ_*i<*_ *j* max (*b*_*ij*_,*b*_*ji*_)] of the multiple-site dissimilarity are analogous to the components *b* and *c* of pairwise dissimilarity, respectively. The fraction [Σ_*i*_ *S*_*i*_ − *S*_*T*_] analogous to the component *a* of pairwise dissimilarity (i.e. the species shared between both sites).

We also calculated the Jaccard pairwise dissimilarity indices proposed by Baselga [19] and Carvalho et al. [14] for comparison to assess the robustness of our results. We found overall beta diversity and its components had almost identical results for all three partitioning methods. A comprehensive review of these methods is out of the scope of this paper, but see [15, 63].

### Data Analyses

#### Nestedness Analysis

We estimated nestedness of breeding bird and lizard communities using a nestedness metric: NODF (Nestedness metric based on Overlap and Decreasing Filling) [64]. NODF is generally considered to have better statistical properties, compared to matrix temperature [65] or discrepancy metric [66]. For example, NODF avoids overestimating nestedness (type I errors), and allows deconstructing total nestedness (NODF) into the independent contributions of columns (NODFc) and rows (NODFr) (i.e. sites and species) to the nested patterns [64, 67, 68]. We calculated the NODF indices with the program NODF version 2.0, and generated null communities using 1000 random simulated matrices based on proportional-row and proportional-column (PP) algorithms [64, 69, 70]. PP algorithm is considered as the preferred model when research systems contain relatively small islands and the scale of analysis is small (e.g. Thousand Island Lake) [68, 69].

In our study, we aimed to investigate whether sites with poorer in species are subsets of sites with richer in species, so we reported the indices of NODF for sites in our study. In addition, we found no lizard species on eight islands after intensive survey, and thus excluded these islands’ communities from our analyses (Table 1).

#### Species Richness Modelling

We tested the relationships among island variables using pairwise Pearson correlation coefficients (*r*). Species richness on islands was regressed against island area, isolation, and habitat richness for breeding birds and lizards using linear regression. We used backward stepwise regression to choose the best-fitted model with variable selection and model evaluation based on the Akaike information criteria (AIC) [71]. All island variables were log-transformed to normalize model residuals.

#### Beta Diversity Patterns

To make comparable dissimilarities computed for breeding bird and lizard communities with different numbers of islands (bird 37 islands vs. lizard 29 islands), we computed dissimilarity values for breeding bird communities using a resampling procedure, taking 100 random samples of 29 inventories and computing the average dissimilarity values [19]. We then obtained the proportion of nestedness-resultant component to overall multiple-site dissimilarity to represent the relative contribution of overall beta diversity: β_ratio_ = β_SNE_/β_SOR_. Thus, β_ratio_ < 0.5 indicates that beta diversity is determined dominantly by species turnover, and β_ratio_ > 0.5 indicates nestedness is the dominant component [24].

We tested whether differences in island attributes (area, isolation and habitat richness) had significant relationships with bird and lizard beta diversity and their components. The pairwise dissimilarities between islands are not independent because the species composition of one island affects the dissimilarity of this island with all other islands. We thus used multiple regression models for distance matrices (MRM) to examine the relationships between the matrices of overall beta diversity, turnover and nestedness-resultant dissimilarities and the Euclidean distance matrices of environmental variables [22, 72, 73]. We then obtained the regression slopes (*a*) and intercepts (*b*) by MRM. Because non-independent observations of pairwise distances will inflate the significance of statistical tests, and our analyses could be affected by spatial autocorrelation, we used partial Mantel tests (9999 permutations) including spatial distance between islands as a covariate to estimate the *p*-values and the Pearson correlation coefficients (*r*) [72]. In our study, the regression slopes (*a*) and intercepts (*b*) of β_sor_ equated the summations of β_sim_ and β_sne_, respectively because of the property of the additive partitioning method.

We performed statistical analyses in R [74] using packages *ape* [75], *betapart* [76], *ecodist* [77] and *vegan* [78], and NODF program (version 2.0) [70].

## Results

### Nestedness Structure

The observed NODF for sites of breeding birds (N_obs_ = 81.66) was significantly lower than expected from the null model (N_exp_ = 85.38, *Z*-value = −2.49, *p* = 0.01), whereas the observed NODF for sites of lizards (N_obs_ = 62.73) was not significantly different (N_exp_ = 61.70, *Z*-value = 0.39, *p* = 0.35) (Table 2). It indicated breeding bird communities were significantly anti-nested (i.e. observed community less nested than expected by null matrices), and lizard communities were not significantly more nested than random patterns. S1 and S2 Figs showed the maximally packed matrix for breeding birds and lizards, respectively.

**Table 2:** Results of nestedness analysis using NODF program for species by site matrix of breeding bird and lizard communities on 37 study islands in the Thousand Island Lake, China.

Speicies	Number of species	Number of island	N_obs_	N_exp_ (SD)	Filling	*Z*-value	*p*
Breeding birds	60	37	81.66	85.38 (1.49)	41.0%	–2.49	0.01
Lizards	5	29	62.73	61.70 (2.66)	40.0%	0.39	0.35

Islands with no lizard species (*N* = 8) were excluded from the analysis. Null model was based on proportional-row and proportional-column constrains with 1000 randomizations. Abbreviations: observed NODF for sites, N_obs_; expected NODF for sites with standard deviation, N_exp_ (SD); *Z*-value; Monte Carlo-derived probabilities, *p*.

### Patterns of Species Richness

Island area was significantly correlated with habitat richness (*r* = 0.89, *p* < 0.05), indicating that larger islands generally support more habitat types. There were no significant correlations between isolation and island area or habitat richness.

Species richness patterns of breeding birds and lizards are similar as shown by the linear regression models and stepwise analyses: only island area was retained in the best-fitted models and was positively related to species richness of both groups (Table 3).

**Table 3:** Linear regression models and stepwise regressions of species richness on island attributes (island area, isolation, and habitat richness) for breeding birds and lizards on 37 study islands in the Thousand Island Lake, China.

Species richness	Variables	Estimate	Standard Error	*R* ^2^	*p*
Breeding birds	Intercept	1.34	0.01		< 0.05
	Area	0.09	0.01	0.75	< 0.05
Lizards	Intercept	0.09	0.03		< 0.05
	Area	0.24	0.03	0.70	< 0.05

Only island variables in the best-fitted model were shown.

### Multiple-Site Dissimilarities

**Fig 2:**
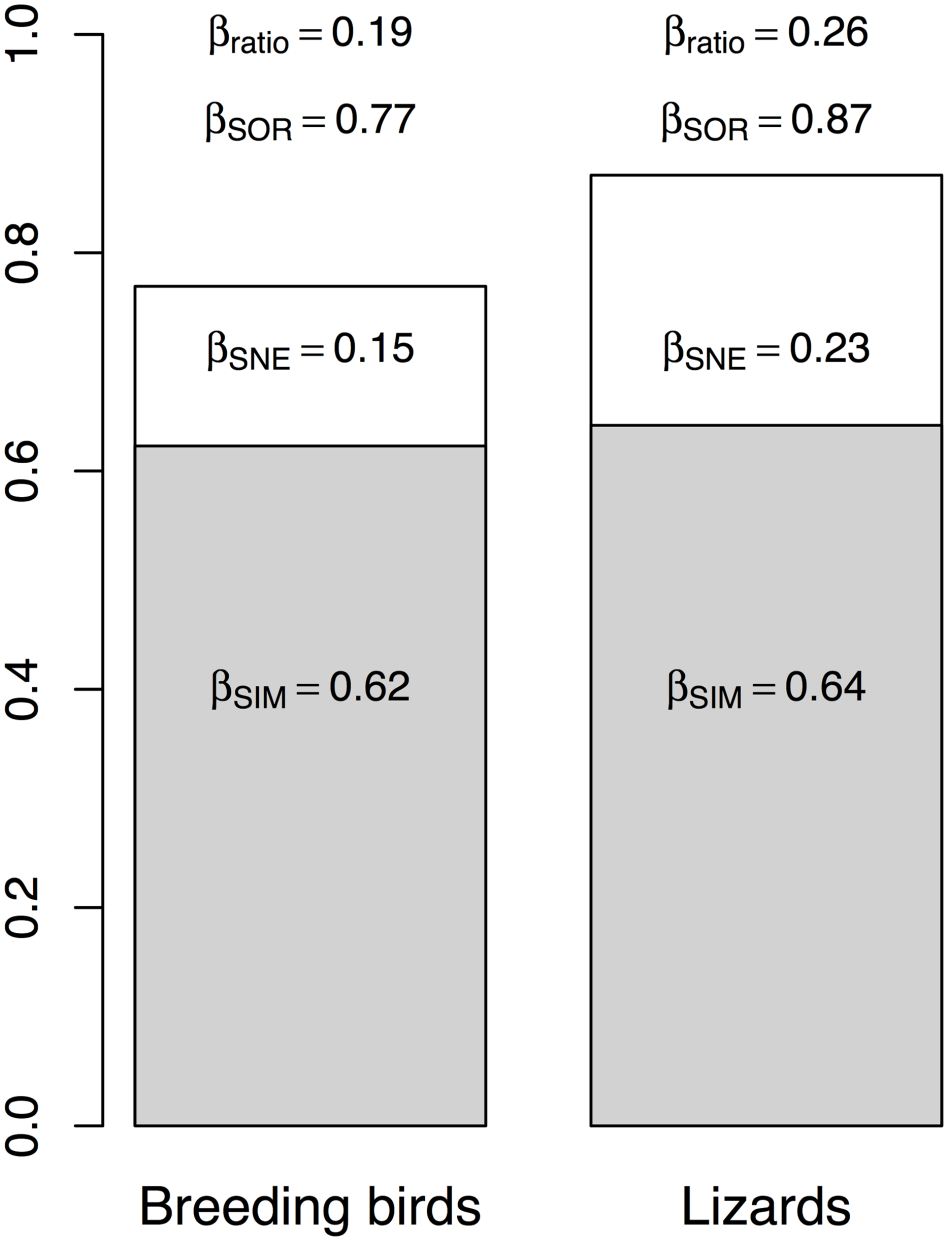
The multiple-site Sørensen dissimilarity (β_SOR_) and its components of turnover (β_SIM_) and nestedness-resultant (β_SNE_) of breeding bird and lizard communities on 37 study islands in the Thousand Island Lake, China. β_ratio_ indicates the ratio of β_SNE_ to β_SOR_.

Multiple-site Sørensen dissimilarity among all study islands was relatively lower for breeding birds than for lizards (0.77 vs. 0.87, respectively). The spatial turnover component was the largest fraction of overall dissimilarity, and reached similar values in birds and lizards (0.62 vs. 0.64). Therefore, the difference in overall beta diversity of breeding bird and lizard communities was caused by variation in the nestedness-resultant component (0.15 vs. 0.23) (Fig 2). Because of the larger turnover values, the ratio of nestedness-resultant component to beta diversity was < 0.5 in both groups, and lower in birds (β_ratio_ = 0.19) than in lizards (β_ratio_ = 0.26). The low β_ratio_ values indicated that variation of community compositions of breeding birds and lizards were predominantly related to spatial turnover.

### Pairwise Dissimilarities

**Fig 3:**
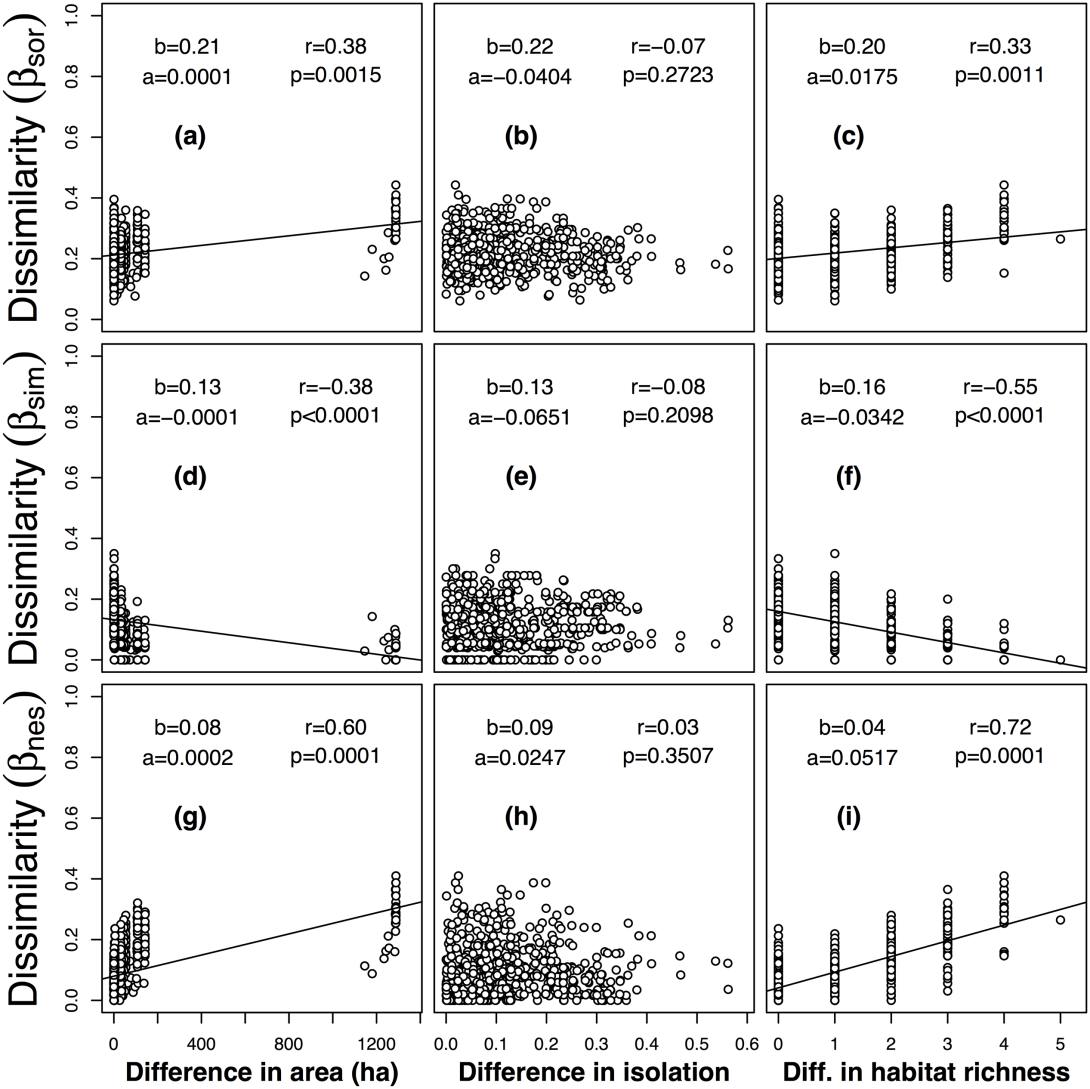
The relationship of overall beta diversity (β_sor_) and its components of turnover (β_sim_) and nestedness (β_sne_), with differences in island area, isolation and habitat richness of breeding bird communities on 37 study islands surveyed from 2007 to 2012 in the Thousand Island Lake, China. Abbreviations: slope of multiple regression model, *a*; intercept of multiple regression model, *b*; Pearson correlation coefficient, *r*; *p*-value of Mantel permutation test, *p*.

No significant relationships existed between difference in isolation and pairwise dissimilarities of breeding bird communities (Fig 3b, 3e and 3h). The turnover component decreased significantly with difference in area (*r* = −0.38, *p* < 0.05; Fig 3d), and difference in habitat richness (*r* = −0.55, *p* < 0.05; Fig 3f), respectively. The nestedness-resultant component increased significantly with difference in area (*r* = 0.60, *p* < 0.05; Fig 3g), and difference in habitat richness (*r* = 0.72, *p* < 0.05; Fig 3i), respectively. Overall dissimilarity increased significantly with difference in area (*r* = 0.38, *p* < 0.05; Fig 3a), and difference in habitat richness (*r* = 0.33, p < 0.05; Fig 3c), respectively.

**Fig 4:**
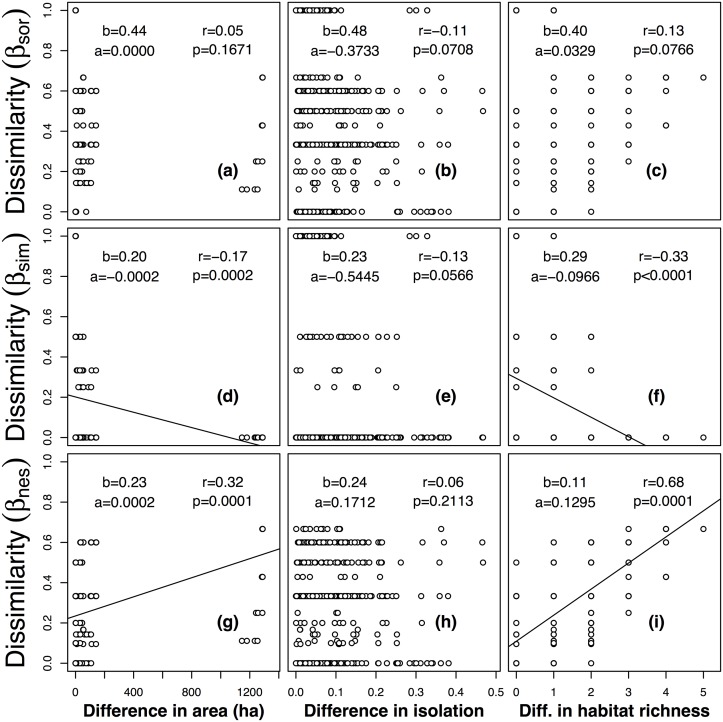
The relationship of overall beta diversity (β_sor_) and its components of turnover (β_sim_) and nestedness (β_sne_), with differences in island area, isolation and habitat richness of lizard communities on 37 study islands surveyed from 2007 to 2008 in the Thousand Island Lake, China. Abbreviations: slope of multiple regression model, *a*; intercept of multiple regression model, *b*; Pearson correlation coefficient, *r*; *p*-value of Mantel permutation test, *p*.

In general, lizard communities showed similar relationships between dissimilarities and differences in island attributes. There were no significant relationships between difference in isolation and pairwise dissimilarities (Fig 4b, 4e and 4h). The turnover component also decreased significantly with difference in area (*r* = −0.17, *p* < 0.05; Fig 4d), and difference in habitat richness (*r* = −0.33, *p* < 0.05; Fig 4f), respectively. The nestedness-resultant component also increased significantly with difference in area (*r* = 0.32, *p* < 0.05; Fig 4g), and difference in habitat richness (*r* = 0.68, *p* < 0.05; Fig 4i), respectively. In contrast with breeding bird communities, however, overall pairwise dissimilarity of lizard communities had no significant relationship with differences in area, isolation and habitat richness, respectively.

## Discussion

### Multiple-Site Dissimilarities

As shown by NODF results (Table 2), breeding bird communities were significantly anti-nested, whereas lizard communities were not significantly more nested than random patterns—neither of them was significantly nested. We also found that the value of nestedness (NODF for sites) of lizards (62.73) was smaller than breeding birds (81.66), whereas the nestedness-resultant component of lizards (0.23) was larger than breeding birds (0.15). It should be noted that β_SNE_ index is not a measure of nestedness itself, but a measure of how different are sites because of nestedness [9]. Therefore, β_SNE_ depends both on the nestedness (i.e. NODF) and species richness differences. In our study, the richness differences in lizards (coefficient of variation of species richness, CV = 0.61) were much larger than in birds (CV = 0.21). Thus, although nestedness of breeding bird communities was higher than that of lizard communities, because of the much larger richness differences in lizards, the dissimilarity due to nestedness (β_SNE_) was larger in lizards than in breeding birds.

The β_ratio_ indices of breeding birds and lizards were both low in our system (0.19 for birds, and 0.26 for lizards), indicating that turnover was the dominant contributor to beta diversity. In our study system, the vast majority of islands are relatively small (≈ 1 ha) [48] (see also Fig 1), and some bird species on these small islands were exclusive to them compared with other small islands with similar areas. It indicates that composition changes among islands mostly because of species replacement (moderate species turnover), and less importantly because of differences in richness (small nestedness-resultant component). Another possible explanation might be the non-nestedness patterns in our research system, especially the anti-nestedness structures of breeding bird communities. Anti-nestedness might hypothetically relate to assembly rules mediated through competitive interactions that may generate differences in community composition from island to island, and thus potentially reduce nestedness in our land-bridge island system [68, 79, 80].

The lower β_ratio_ index of breeding birds is in accordance with our prediction that species with stronger dispersal ability will be less affected by barriers, and will be able to occupy most of the appropriate habitats in a region [81, 82]. Moreover, the higher multiple-site nestedness-resultant dissimilarity of lizards suggests a higher probability of local extinction derived from habitat fragmentation. The rationale behind this inference is that, because pairwise nestedness-resultant dissimilarity is positively associated to differences in area, small islands have suffered more species losses [11, 31]. Given that the multiple-site nestedness-resultant dissimilarity is higher in lizards, this suggests that sequential local extinctions linked to nested patterns have more relevance in lizards than in birds. An alternative interpretation of the lower β_ratio_ of breeding birds is that such species loss on small islands may be systematic (deterministic), rather than stochastic, which may dependent on some species traits, e.g. the degree of generalism and migration capacity of the species [83–86]. These in turn will need studies with detailed resource and migration information to test these hypotheses.

### Pairwise Dissimilarities

In our study, we found that neither turnover nor nestedness-resultant components had significant relationships with difference in isolation for breeding bird and lizard communities. In turn, nestedness-resultant components of both breeding birds and lizards increased significantly with differences in area and habitat richness, and spatial turnover had the opposite trend. As a result, the patterns that overall pairwise dissimilarity of breeding birds and lizards increases with differences in area and habitat richness were driven by the nestedness-resultant component, and not by spatial turnover components.

Regarding island isolation, geological barriers mainly affect animals with poorer dispersal ability, e.g. the open water surface in our system. Compared with oceanic islands, the relative small scale of our research system associated with the high mobility of birds diluted the biological importance of isolation [2, 48, 87]. Furthermore, the water surface between nearby islands in the lake does neither seem to be a barrier for lizards, because we did not detect the effects of dispersal limitation on dissimilarities (Fig 4e–4h). It also means selective colonization may also play no role in breeding bird and lizard distributions on the islands [43].

As for island area, the main mechanism influencing community composition is selective extinction. Because all previous mountaintops in the lake region were continuous, the water level increased rapidly when the dam constructed, and isolated the mountaintops that became the islands. Animals that were sensitive to habitat loss were locally extinct on small islands, but their populations persisted in long term on larger islands. Because of species loss, assemblages on smaller islands tend to be a subset of assemblages on larger islands, resulting in nested patterns [43, 88, 89]. Our results showed that nestedness-resultant dissimilarity increased, and turnover decreased respectively when difference in area increased (Figs 3g, 3d, 4g and 4d). In other words, higher nestedness-resultant dissimilarity existed on islands with large difference in area (also as shown by species richness modeling; Table 3). However, higher turnover existed on islands with similar areas and, as a result, species replacement contributed mostly to overall beta diversity. This result is consistent with the hypothesis of selective extinction, in accordance with previous studies in the same system [43].

Finally, regarding habitat richness, turnover and nestedness-resultant components of beta diversity of breeding birds and lizards followed the same patterns as difference in area: turnover decreased, and nestedness increased with increasing difference in habitat richness, respectively. This can be interpreted as turnover being higher on islands with similar number of habitats (Figs 3f and 4f), while islands with larger difference in habitat richness were prone to have higher nestedness-resultant components (Figs 3i and 4i). In our study system, island area is positively correlated with habitat richness, and the effect of habitat richness may still more important than area both for breeding birds and for lizards, as indicated by the largest correlation coefficient between the nestedness-resultant component and difference in habitat richness (*r* = 0.72 for birds vs. 0.68 for lizards).

## Conclusion

Partitioning beta diversity of breeding bird and lizard communities into turnover and nestedness-resultant components in the Thousand Island Lake, China, revealed that overall beta diversity of lizard communities in our system was relatively higher than breeding bird communities, and spatial turnover contributed dominantly to beta diversity in both groups. Pairwise dissimilarities of breeding birds and lizards both increased significantly with differences in area and habitat richness. Neither turnover nor nestedness-resultant components of breeding birds and lizards had relationships with difference in isolation, whereas spatial turnover component decreased, and nestedness-resultant component increased with differences in area and habitat richness, respectively. The dominance of the spatial turnover component suggests that all islands have potential conservation value. If a subset of islands is to be prioritized, we then recommend selecting the large islands plus a subset of small islands with high levels of spatial turnover to represent all species. However, it should be stressed that we identified communities on islands as conservation unities, so we considered the conservation value of all islands from the perspective of assemblages, without weighting particular species based on their specific conservation status. In addition, multiple-site dissimilarity values in our analyses cannot be interpreted in absolute terms, as they are dependent the number of sites. In fact, based on pairwise dissimilarity values, assemblage heterogeneity seems low to moderate in both birds and lizards.

## Supporting Information

S1 Dataset The distribution matrix of breeding bird communities by species (columns) × islands (rows) on 37 study islands in the Thousand Island Lake, China. Refer to Fig 1 for the island codes.
(CSV)

S2 Dataset The distribution matrix of lizard communities by species (columns) × islands (rows) on 37 study islands in the Thousand Island Lake, China. Refer to Fig 1 for the island codes.
(CSV)

S1 Fig The maximally packed matrix for breeding bird communities on 37 study islands surveyed from 2007 to 2012 in the Thousand Island Lake, China. Rows: species (*N* = 60); columns: island (*N* = 37, island codes as in Fig 1); shaded cells: species present; unshaded cells: species absent.
(PDF)

S2 Fig The maximally packed matrix for lizard communities on 37 study islands surveyed from 2007 to 2008 in the Thousand Island Lake, China. Islands with no lizard species (*N* = 8) were excluded from the analysis. Rows: species (*N* = 5); columns: island (*N* = 29, island codes as in Fig 1); shaded cells: species present; unshaded cells: species absent.
(PDF)
