# Supplementary figures and images for: Revealing Beta-Diversity Patterns of Breeding Bird and Lizard Communities on Inundated Land-Bridge Islands by Separating the Turnover and Nestedness Components
Source: PLoS One. 2015 May 18;10(5):e0127692. doi: 10.1371/journal.pone.0127692 (PMC4436251; doi:10.1371/journal.pone.0127692)

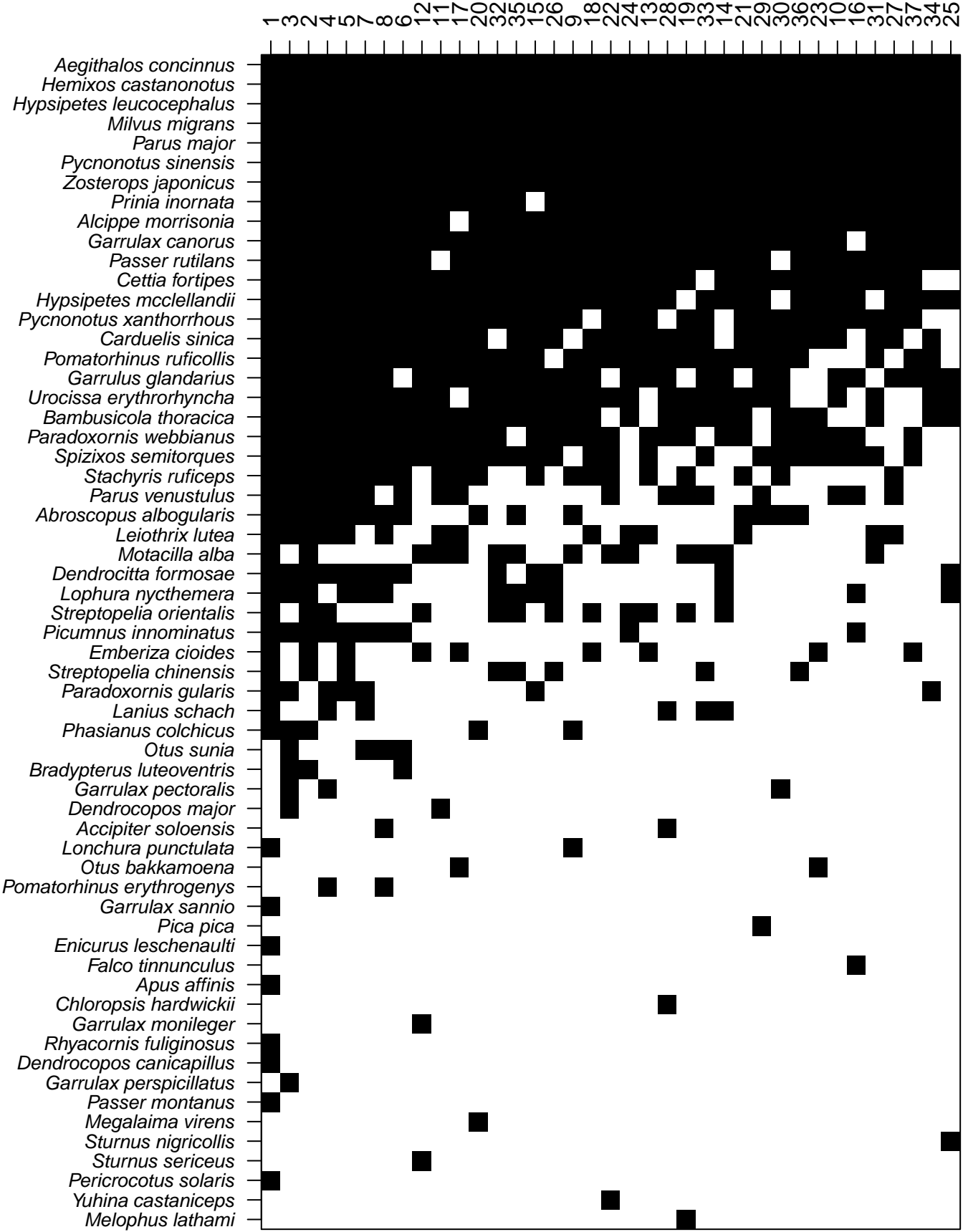

Supplement: S1 Fig — Rows: species (N = 60); columns: island (N = 37, island codes as in Fig 1); shaded cells: species present; unshaded cells: species absent. (PDF) [file pone.0127692.s003.pdf]

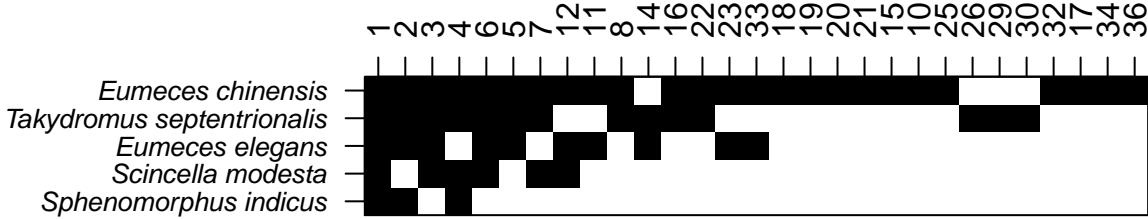

Supplement: S2 Fig — Islands with no lizard species (N = 8) were excluded from the analysis. Rows: species (N = 5); columns: island (N = 29, island codes as in Fig 1); shaded cells: species present; unshaded cells: species absent. (PDF) [file pone.0127692.s004.pdf]
